# Disentanglement of growth dynamic and thermodynamic effects in LaAlO_3_/SrTiO_3_ heterostructures

**DOI:** 10.1038/srep22410

**Published:** 2016-03-24

**Authors:** Chencheng Xu, Christoph Bäumer, Ronja Anika Heinen, Susanne Hoffmann-Eifert, Felix Gunkel, Regina Dittmann

**Affiliations:** 1 Peter Grünberg Institute 7, Forschungszentrum Jülich GmbH, 52425 Jülich, Germany; 2 Institute of Materials in Electrical Engineering and Information Technology II, RWTH Aachen University, 52074 Aachen, Germany

## Abstract

The influence of non-equilibrium and equilibrium processes during growth of LaAlO_3_/SrTiO_3_ (LAO/STO) heterostructures is analyzed. We investigate the electronic properties of LAO/STO heterostructures obtained at constant growth conditions after annealing in different oxygen atmospheres within the typical growth window (1 × 10^−4^ mbar –1 × 10^−2^ mbar). The variation of annealing conditions is found to cause a similar change of electronic properties as observed for samples grown in different oxygen pressure. The results indicate that equilibrium defect formation is the dominant process for establishing the properties of the two-dimensional electron gas (2DEG), while growth dynamics play a minor role in the typical LAO/STO growth regime. Furthermore, the effects of non-equilibrium processes occurring during growth are investigated in detail by quenching just-grown LAO/STO heterostructures directly after growth. We show that during growth the sample is pushed into a non-equilibrium state. After growth, the sample then relaxes towards equilibrium, while the relaxation rate strongly depends on the ambient pressure. The observed relaxation behavior is mainly associated with a reoxidation of the STO bulk, while the 2DEG is formed immediately after the growth.

The discovery of the conductive interface between the two band insulators LaAlO_3_ (LAO) and SrTiO_3_ (STO)^1^,^2^ has led to tremendous efforts in the scientific community to understand the mechanisms for the formation of the electron gas and furthermore to gain control over its electronic properties. The unique properties of these electron gases, such as high mobility^1^,^3^, electric field-control^4^, superconductivity^5^, and magnetism^6^,^7^,^8^ bear exciting potential both for fundamental research^9^ as well as for novel oxide electronic applications^10^,^11^,^12^,^13^.

One major challenge is the great sensitivity of the sample properties on the actual (defect) chemistry at the interface and in the involved bulk materials. This high sensitivity results from the delicate link between chemical composition^14^, ionic defect structure and material properties in complex oxides^15^,^16^. The reported properties of LAO/STO interfaces vary strongly with the particular growth conditions used. Therefore, samples of different research groups are often hardly comparable^1^,^6^,^17^,^18^,^19^. As a result, uncertainties remain about what interface properties are intrinsic and what properties are specific to a certain growth process.

As LAO/STO is almost exclusively obtained by pulsed laser deposition (PLD), at the heart of this problem is the fact that PLD growth is a non-equilibrium process. A sample is typically pushed away from thermodynamic equilibrium during the growth process^20^. It is unclear, however, how far it is pushed away from equilibrium depending on growth conditions, and if and how fast the system relaxes back towards equilibrium after the growth process. For this reason, post-deposition treatments and even cooling rates can have large impact on the established sample properties. As a matter of fact, growth-induced non-equilibrium processes and thermodynamic processes happen simultaneously. Therefore, it is generally difficult to distinguish the effects of either of the two.

One prominent example for this is the change of the temperature dependence of the interfacial resistance of LAO/STO when the growth pressure is varied^1^,^6^. This effect has been attributed to magnetic defects formed during growth, whereas their origin is still under debate^6^. The growth pressure controls the oxidation state of the incoming plasma particles^21^,^22^, the number and impact of scattering processes during plasma propagation^23^,^24^,^25^,^26^ as well as surface diffusion^27^– in other words the entire growth dynamics may be changed upon a change in growth pressure. In addition, the ambient oxygen pressure – being a state variable – is influential to the thermodynamic equilibrium state of the established heterostructure, too^28^,^29^,^30^,^31^,^32^,^33^. Therefore, it is an open question whether the observed change in electrical properties is governed by growth dynamics or by thermodynamics.

Furthermore, it has been observed that the interfacial electron gas is often accompanied by parallel bulk conduction in the STO substrate. This bulk conduction is attributed to a reduction of the STO substrate generated by or during the growth process and appears primarily at low growth pressures. However, the threshold pressure for bulk reduction reported in the literature varies^1^,^6^,^17^,^18^,^19^,^34^,^35^,^36^. Hence, there seems to be an inherent dependence of this bulk reduction on the experimental procedure as well as on the remaining growth conditions such as layer thickness^37^,^38^,^39^ and laser fluence^37^,^40^,^41^.

In the picture of defect chemistry, these phenomena might be well described through the interplay between different defect types. As an intuitive description, the reduction of STO is predominantly related to the generation of oxygen vacancies, which act as donors and generate delocalized electrons in addition to the 2DEG electrons at the interface. Moreover, different experimental setups may result in the generation of other defects that can compensate delocalized electrons, such as intrinsic defects like cation vacancies. Therefore, one has to take the defect chemistry into account when designing the experiment and consider the impact of defects generated during and after PLD deposition.

In this paper, we separate the effects from growth dynamics and from thermodynamics on the defects in the heterostructures by studying the properties of LAO/STO samples that have been grown under identical growth conditions (hence, the growth dynamics are unchanged), and then post-annealed in varied atmospheres within the typical PLD growth window (10^−4^ mbar–10^−2^ mbar). The results indicate that the thermodynamics rather than the growth dynamics yield the variation of the electronic properties of the LAO/STO interface.

Furthermore, we address the non-equilibrium effects during growth, by characterizing the quenched state of just-grown heterostructures. We then follow the relaxation from non-equilibrium towards equilibrium inside the PLD chamber. For this approach, we use a dedicated diode laser heating system which allows to quickly quench samples and its actual non-equilibrium state at a defined point of time after PLD growth. From the experiments, we are able to extract the reoxidation dynamics of the sample as a function of the ambient physical oxygen pressure. We find that just-grown samples exhibit a strongly reduced STO substrate far from thermodynamic equilibrium. After growth, the sample reoxidizes inside the PLD chamber at a rate that strongly depends on the physical oxygen pressure.

## Results

### Impact of Interfacial Thermodynamics

**Figure 1:**
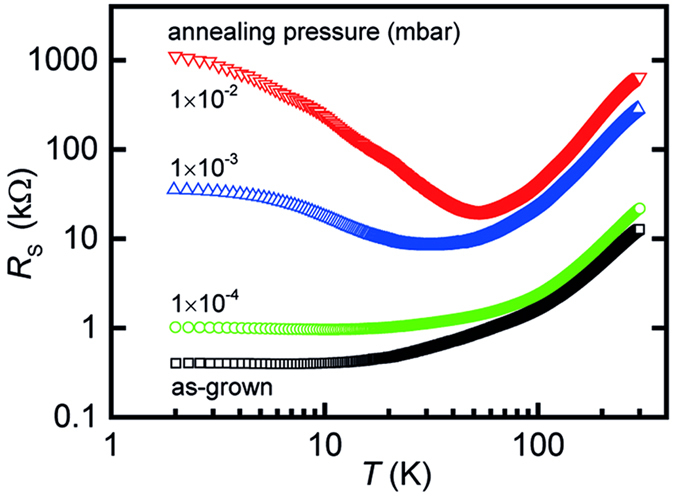
Sheet resistances of LAO/STO heterostructures deposited with T = 700 °C and *p*O_2 _= 1 × 10^−4 ^mbar. The samples were post-annealed at 700 °C in varying *p*O_2_ for one hour.

Figure 1 shows the temperature dependence of the sheet resistance, *R*_S_, of LAO/STO heterostructures grown at a temperature of 700 °C and a growth pressure of 1 × 10^−4^ mbar, corresponding to typical growth conditions resulting in conducting LAO/STO interfaces. All samples have been established in a similar growth process, so that the growth dynamics as well as the thin films’ stoichiometry^14^ are identical for all samples. We compare an as-grown sample with samples that have been post-annealed for one hour at 1 × 10^−4^ mbar (identical to growth pressure), 1 × 10^−3^ mbar, and 1 × 10^−2^ mbar. All samples are cooled down slowly in the indicated atmosphere at a constant rate of 10 °C/min.

The as-grown sample shows metallic behavior with a sheet resistance of about 10 kΩ at room temperature. With decreasing temperature, *R*_S_ decreases constantly saturating at a residual resistance of about 400 Ω at temperatures below 10 K. The sample post-annealed for 1 hour at growth pressure (1 × 10^−4^ mbar), shows a slightly increased sheet resistance at room temperature as well as in the low temperature regime. At low temperature, the samples annealed at low oxygen pressure exhibit a non-linear Hall-effect^42^ indicating multi-channel conduction^43^,^44^. The high-mobility channel exhibits a mobility of about 2000 cm^2^/Vs in accordance with the literature under similar deposition condition^17^,^43^,^44^(see SI Fig. 2). At increased annealing pressure (1 × 10^−3^ mbar), the room temperature sheet resistance is further increased (*R*_S_ ≈ 100 kΩ). Moreover, the temperature dependence of *R*_S_ now exhibits a clear (Kondo-like^6^,^45^) minimum at about 30 K, reaching a residual resistance of 35 kΩ at 2 K. The sample annealed at 1 × 10^−2^ mbar shows a similar behavior, while the resistance characteristics is further shifted towards higher values. A resistance minimum is found at 60 K, and the resistance at 2 K corresponds to about 1 MΩ.

The identical behavior of the sheet resistance has been reported in the literature^6^,^19^,^37^,^46^ for LAO/STO samples that are *grown* at pressures similar to the ones used here for the *post-annealing* process. Hence, it does not seem to be important at what oxygen pressure the LAO/STO interface has been formed, but it is important at what *p*O_2_ the established structure has been equilibrated and cooled down. It is thus evident that rather equilibration with the ambient atmosphere is decisive for the low temperature electronic properties of the LAO/STO interface, while the altered growth dynamics have negligible effect (provided that the film quality is good enough to form a conducting interface at all).

As shown in the literature^6^, the increase in sheet resistance with increasing oxygen partial pressure is mainly caused by a decrease in electron mobility, indicating that *additional* defects are induced at the interface during annealing and equilibration at increased *p*O_2_. Therefore, oxygen vacancies are unlikely to be the relevant defect species here, as their concentration should be reduced at increased *p*O_2_. In contrast, the associated thermodynamic driving force during equilibration has been identified as the Schottky-equilibrium^28^,^47^,^48^ describing the balance of electronic and ionic charge compensation at the interface. This balance shifts towards ionic charge compensation at increased oxygen pressure, so that the observed dependence of *R*_S_ on *p*O_2_ can be interpreted as indicative for the insertion of cation vacancies in the near-interface region – in particular Sr vacancies as a result of the high formation energy of Ti vacancies^49^,^50^.

### Impact of Growth dynamics

Due to the post annealing and the slow cooling procedure, all samples discussed so far remain at or near the growth temperature for considerably long times (>30 minutes above 400 °C). They may thus be considered as close-to-equilibrium samples. In the second part of this paper, we now turn to the impact of non-equilibrium effects during PLD growth. We characterize the non-equilibrium state of the just-grown heterostructures, illustrate the relaxation process of the as-grown samples from non-equilibrium back into their equilibrium states inside the PLD chamber, and discuss the kinetics of the associated processes.

For this, we fabricate two sequences of samples, which are deposited at 800 °C and a growth pressure of 1 × 10^−3^ mbar and 1 × 10^−4^ mbar, respectively. After growth, the samples are kept at growth pressure and temperature for a defined duration, *t*_a_, ranging from 0 s (quenching immediately after the growth) up to 3600 s (post-deposition annealing for 1 h).

Being equipped with a diode laser heating system, the PLD system in use allows us to quickly cool down the sample (quenching) by switching off the diode laser. The typical cooling rate is about 10 °C/s (above 400 °C), i.e. about 60 times faster than in the experiments described above. In this way, the defect state obtained after growth is conserved, as ionic motion is immediately suppressed upon cooling.

**Figure 2:**
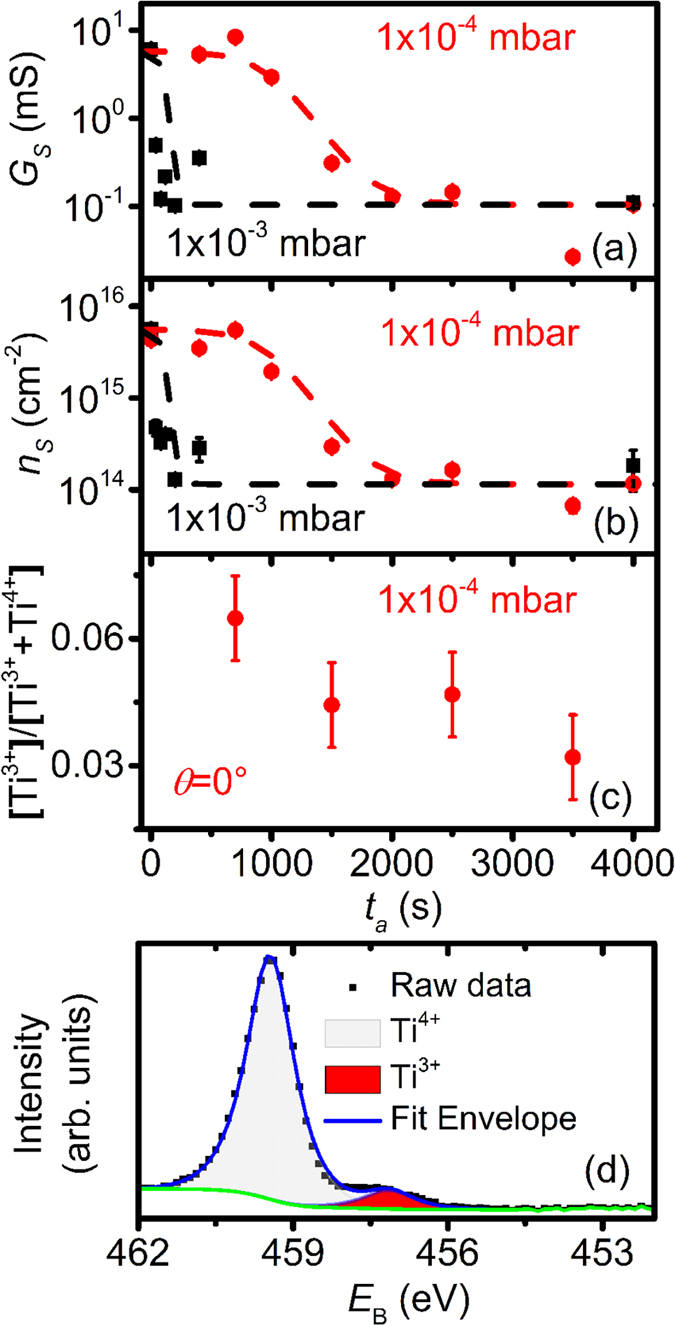
Transient properties of LAO/STO heterostructures after PLD growth. (**a**) Room temperature sheet conductance, *G*_S_, of LAO/STO samples quenched at a defined time after growth. (**b**) Corresponding sheet carrier density, *n*_S_. (**c**) Ti^3+^ fraction obtained from XPS spectra (principle depicted in (**d**)) recorded on samples quenched after various periods of time. The solid lines are guide for the eyes.

Figure 2a shows the room temperature sheet conductance, *G*_S_, of LAO/STO heterostructures measured *ex-situ* after annealing for various *t*_a_. The corresponding sheet carrier densities, *n*_S_, are presented in Fig. 2b.

For both growth pressures, the conductance measured for samples quenched immediately after growth (*t*_a_ ≈ 0 s) is by almost two orders of magnitude larger than the conductance measured for samples quenched after annealing for one hour (*t*_a_ ≈ 3600 s). (The initial values are also much larger than the room temperature conductance of the samples cooled down slowly as shown in Fig. 1). For intermediate *t*_a_, the conductance relaxes from its high value obtained directly after growth towards its lower (equilibrium) value obtained after one hour and remains unchanged upon further annealing. We thus monitor how the as-grown LAO/STO heterostructures transit from a non-equilibrium state (varying in time) to their equilibrium state (stable over time) inside the PLD chamber. The relaxation rate strongly depends on the actual *p*O_2_. In particular, equilibrium is reached after ~100 s at 1 × 10^−3^ mbar (black symbols) and after ~1800 s at 1 × 10^−4^ mbar (red symbols), respectively.

As shown in Fig. 2b, the sheet charge carrier density shows a similar trend as observed for the conductance. While *n*_S_ is of about 5 × 10^15^ cm^−2^ for *t*_a_ = 0 s, it relaxes to an equilibrium value of about 1 × 10^14^ cm^−2^ at *t*_a_ = 3600 s.

This equilibrium value is a typical carrier density reported for the interfacial 2DEG^1^,^17^,^19^. The non-equilibrium value, however, is too large to be accommodated at the confined interface only (it would corresponds e.g. to a volume concentration of 1 × 10^22^ cm^−3^ ≈ 60 at% considering a 2DEG thickness of about 5 nm). Therefore, the high concentration of electrons observed immediately after the growth has to be attributed to a bulk conduction effect inside the STO substrate. In other words, during the growth process the STO substrate is strongly reduced, resulting in the formation of oxygen vacancies inducing conduction in the bulk of STO.

As a consequence, the relaxation process observed for various *t*_a_ (Fig. 2a,b) has to be attributed to the reoxidation of the bulk of the STO substrate inside the PLD chamber. Thus, this reoxidation process is an additional relaxation process occurring after PLD growth of LAO/STO heterostructures, superimposed by the establishing of the Schottky-equilibrium in the near-interface region (see Fig. 1).

Our finding is in good accordance with the phenomena observed by Basletic *et al.*^36^ with conductive AFM for the bulk and interface properties in LAO/STO system, where the interface of LAO/STO remains almost unchanged after post annealing process and the charge carrier concentration in the STO bulk decreases over 2 orders of magnitude because of reoxidation processes during post annealing.

Our results do not only verify the existence of this oxidation process, but also allow us to study the pressure-dependent kinetics of this process and to disentangle the effects of growth dynamics and thermodynamics on the interface of LAO/STO.

This reoxidation process can also be visualized and the contributions of interface and bulk conduction can be separated from the Ti^3+^ content determined by XPS (normal incidence, Fig. 2c). For XPS analysis, we use the sample sequence obtained at 1 × 10^−4^ mbar and an LAO layer thickness of 5 unit cells to minimize signal attenuation by the capping layer.

In the XPS spectrum obtained at the Ti 2p3/2 peak, we observe a small shoulder resulting from the presence of Ti^3+^, in addition to the usually observed Ti^4+^ peak (Fig. 2d). Fitting two concentration components reveals a drop in the [Ti^3+^]/[Ti^3+^ + Ti^4+^] ratio, from about 6 at% for *t*_a_ = 0 s to about 3 at% at *t*_a_ = 3600 s.

Based on a model comprising both interface and bulk contributions (see supplementary material), we can attribute this drop by 3 at% to a vanishing bulk contribution, while the interface contribution is constant over time accommodating the entire Ti^3+^ content of about 3 at% found for *t*_a_ = 3600 s. This percentage of Ti^3+^ is in good accordance with the sheet carrier density at *t*_a_ = 3600 s of ~1.2 × 10^14^ cm^−2^ (Fig. 2a). The detailed comparison can be found in supplementary material. The constant interface contribution forms immediately at *t*_a_ = 0 s. The vanishing bulk contribution is associated with the oxidation process of the sample during relaxation from non-equilibrium after the growth to equilibrium, as discussed above.

As a matter of fact, the observation of a reoxidation effect in the bulk of STO indicates that the equilibrium state of STO under typical growth conditions for LAO/STO is a well-oxidized one. The observation of bulk conduction in LAO/STO is hence related to the non-equilibrium into which the growing sample has been pushed during growth.

Interestingly, for both growth pressures the non-equilibrium states obtained directly after growth are almost identical with *G*_S_ ≈ 60 mS (*n*_S_ ≈ 5 × 10^15^ cm^−2^). However, the equilibration process differs depending on ambient pressure and exhibits distinct features. At 1 × 10^−3^ mbar, the reduction of conductance starts immediately as the post-annealing begins. Equilibrium is reached already after a few hundreds of seconds. At 1 × 10^−4^ mbar, annealed samples firstly remain almost unchanged for about 1000 s before the carrier density starts to drop. Moreover, the time needed for the (retarded) conductance equilibration is larger at 1 × 10^−4^ mbar than at 1 × 10^−3^ mbar (cf. slope of dashed lines in Fig. 2a,b which are guides to the eye).

The almost identical initial non-equilibrium states at 1 × 10^−3^ mbar and 1 × 10^−4^ mbar indicate that the reduction of the STO substrate is induced predominantly by the plasma plume dynamics, while the actual partial pressure seems less important. The plasma dynamics are essentially unchanged in the considered pressure range^21^. In particular, the oxidation states of the incoming plasma species are rather similar in this pressure range^21^. Thus, oxygen gettering^3^,^51^ by the growing layer should be similar, too.

In contrast to this, the *p*O_2_ is much more important for reoxidation inside the PLD chamber. The reoxidation happens through two main steps: surface incorporation and bulk diffusion. For specific conditions, either of the two is slower than the other and acts as the rate limiting step. At growth temperature, oxygen vacancy or oxygen ion diffusion is rather fast in the bulk of STO (D_Vö_ ≈ 3 × 10^−6^ cm^2^/s at 800 °C^52^). In contrast to that, the surface incorporation rate of oxygen into perovskite is low (*k*_S_ ≈ 10^−9^ cm/s^53^). Hence, the rate limiting step for the reoxidation process should be surface incorporation. Therefore, the difference in the transient behavior observed for different growth and annealing pressures indicates that the incorporation of oxygen ions into the STO bulk is hindered at a physical pressure of 1 × 10^−4^ mbar, while it is considerably faster at 1 × 10^−3^ mbar. One possible scenario could be a varied surface exchange coefficient, *k*_S_, of the LAO thin film due to the different growth conditions. We explicitly tested this scenario by comparing samples grown even below 1 × 10^−4^ mbar and annealing them in 1 × 10^−3^ mbar, which resulted in a similar equilibration time as observed for the samples grown at 1 × 10^−3^ mbar. Therefore, we can exclude a modification of *k*_S_ at lower *growth* pressure and assign the increased equilibration time to the *annealing* pressure. The observed relaxation behavior is thus consistent with the reduced attempt frequency of oxygen attaching to the sample at 1 × 10^−4^ mbar.

### Disentanglement of growth dynamics and thermodynamics

In order to illustrate how much the initial non-equilibrium state obtained after growth differs from equilibrium, we compare the results of the quenching experiments with the high temperature equilibrium conductance (HTEC) data obtained experimentally for single crystal STO and for LAO/STO heterostructures in our previous work^28^,^31^. A detailed discussion of the oxygen pressure dependence of the conductance can be found within the corresponding publications^28^.

From the comparison of the data, it is directly displayed how the equilibration process observed for LAO/STO heterostructures inside the PLD chamber compares with the equilibrium states probed in ex-situ HTEC experiments. For this, we rescale the Hall data from room temperature (Fig. 2b) to high temperature adopting the temperature dependence of the electron mobility reported in the literature^33^ (for further details see the supplementary materials).

**Figure 3:**
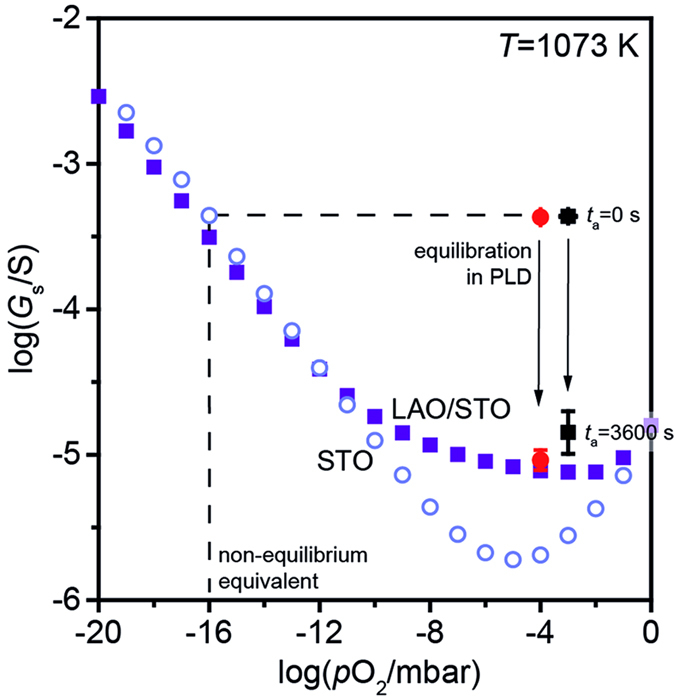
High temperature equilibrium conductance (HTEC) of STO (open symbols) and LAO/STO (closed symbols) at 800 °C between for (−20) < log(*p*O_2_/mbar) < 0. Initial and equilibrated sheet conductance obtained for quenched LAO/STO samples are plotted as black (for 1 × 10^−3^ mbar) and red (for 1 × 10^−4^ mbar) filled symbols. The equilibrium pressure corresponding to the initial quenched state is about 10^−16^ mbar (dashed lines).

Figure 3 shows typical HTEC data as a function of ambient *p*O_2_ obtained at growth temperature (800 °C) for a STO single crystal (open symbols) and a LAO/STO heterostructure (closed symbols), exhibiting an additional, thermally stable conductance contribution observable at intermediate *p*O_2_ values (deviation between open and closed symbols). In addition, we plot the scaled conductance values obtained in this study after quenching directly after growth (*t*_a_ = 0 s) and after 1 hour of equilibration (*t*_a_ = 3600 s).

The initial non-equilibrium states for both pressures correspond to the equilibrated state obtained for LAO/STO at an ambient *p*O_2_ of about 1 × 10^−16^ mbar (dashed lines), which is 12–13 (!) orders of magnitude lower than the nominal growth pressure. Hence, the sample is pushed far away from equilibrium during growth. In this reduced state, the HTEC of LAO/STO heterostructure and STO single crystal overlap, indicating that the non-equilibrium state from PLD is mainly due to the reduced STO bulk. Similarly, a strong reduction of STO caused by PLD plasma is also observed by Scullin *et al.*^20^.

During equilibration (Fig. 2), the conductances of the LAO/STO heterostructures virtually follow the isobaric lines at 1 × 10^−3^ and 1 × 10^−4^ mbar, respectively (black arrow, Fig. 3) drifting towards equilibrium. After *t*_a_ = 3600 s, the equilibrated states of LAO/STO in the PLD chamber are in good accordance with the HTEC data of the LAO/STO 2DEG at 1 × 10^−4^ mbar and 1 × 10^−3^ mbar. At these pressures, the bulk STO yields only a minor contribution to the total HTEC of the LAO/STO heterostructure, confirming that the equilibrated samples exhibit merely interfacial conduction.

Thus, samples equilibrated in growth conditions exhibit negligible contributions of parallel conduction within the substrate. As a consequence, post-deposition annealing at growth pressure is sufficient to suppress the effect of electronic carriers in the bulk of STO. So far, it has been typically assumed that annealing in oxygen-rich atmospheres (10–1000 mbar) is required to achieve a sufficiently insulating STO substrate. However, while such high oxygen pressures may give similar conductance values at room temperature, they may result in considerably increased resistance at low temperature as shown in the first part of the paper (Fig. 1). In particular, higher oxygen pressures during equilibration lead to an increase in residual resistances corresponding to a decrease in electron mobility. This can be attributed to cationic defects existing at the interface (Fig. 1), while the conductance variation at room temperature (Fig. 2) stems mainly from a changing concentration of oxygen vacancies in the bulk. Our results therefore indicate that equilibration at growth pressure is sufficient to suppress bulk contributions to the conductance being beneficial for carrier mobility when compared to post annealing at higher pressures.

Interestingly, the oxygen pressure for which bulk conduction is typically observed in LAO/STO (10^−6^ mbar– 10^−4^ mbar) is a highly oxidizing condition, if compared to typical oxygen partial pressures obtained in chemically controlled reducing atmospheres. For instance, Ar/H_2_ gas mixtures typically deliver *p*O_2_ < 10^−17^ mbar at typical growth temperatures. This emphasizes the crucial role of non-equilibrium effects involved in the growth process for the varying sample properties obtained in different groups. This extreme importance of the non-equilibrium created by PLD is also illustrated clearly through the comparison between non-equilibrated and equilibrated states in Fig. 3.

The reoxidation process triggered in the bulk of STO manifests itself mainly in the carrier density in the substrate’s bulk. As oxygen exchange with the surrounding atmosphere is involved, the equilibration rate strongly depends on ambient pressure. At typical growth pressures, the equilibration time scales from hundreds of seconds to thousands of seconds. This time scale coincides with the typical time scale on which grown samples are still at elevated temperatures after growth in most PLD setups (typically a few minutes). As the electrical properties of LAO/STO heterostructures and in particular the ones of the STO substrate are still very sensitive within this time, kinetic limitations for oxidation may explain the observed scatter in the properties of samples grown at comparable growth conditions by different research groups and setups.

In particular, at a growth pressure of 1 × 10^−4^ mbar, a sample cooled down directly after growth may exhibit significant parallel conduction in bulk, while a sample cooled down slowly or kept at growth conditions for about 30 minutes shows merely interface conduction (such as for the samples discussed in Fig. 1). At a growth pressure of 1 × 10^−3^ mbar, almost any sample cooled down immediately after the growth (unless intentionally quenched within seconds as described here) should exhibit interface conduction only because equilibration is sufficiently fast.

Based on the discussion above, we can suggest a preferred growth recipe for fabricating an electron gas at the LAO/STO interface with minimal scattering centers and without parallel conduction in the bulk: after the typical growth of LAO on STO for a conductive interface (e.g. 1 × 10^−4^ mbar and 800 °C), a post annealing process on the order of one hour should be carried out under growth conditions, rather than with higher oxygen pressures.

## Discussion

**Figure 4:**
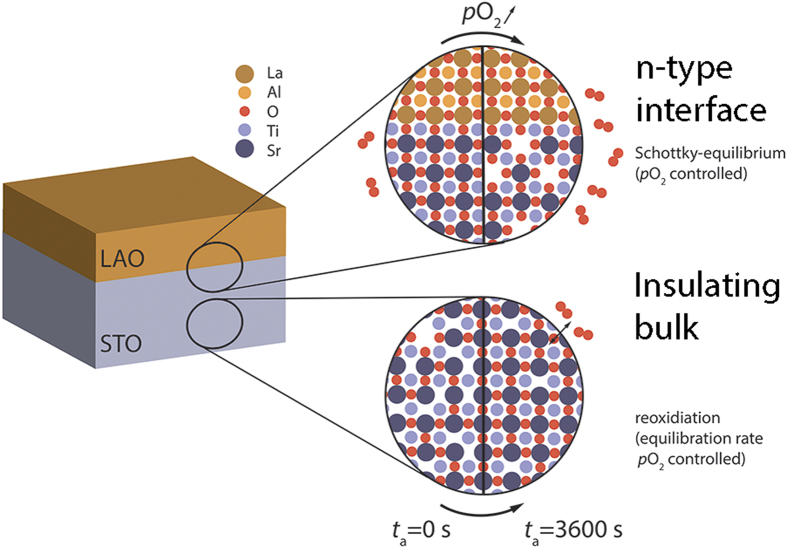
Thermodynamic processes in LAO/STO heterostructures. (1) reoxidation of the STO substrate as acceptor doped bulk after reduction during the PLD process controlled via annealing time, *t*_a_ (bottom); (2) incorporation of strontium vacancies in the n-type near-surface region upon high-pressure annealing controlled via *p*O_2_ (top).

In summary, this study reveals two distinct thermodynamic processes involved in the equilibration of the defect structure of LAO/STO heterostructures after being pushed far away from equilibrium during the PLD process: 1) reoxidation of the STO substrate after reduction during the PLD process; 2) incorporation of strontium vacancies in the near-surface region upon high-pressure annealing (Fig. 4).

While the substrate reduction relies on growth-induced non-equilibrium effects in the bulk, the incorporation of strontium vacancies is related to thermodynamic equilibrium effects in the interface-near region (controlled via *p*O_2_) deriving from the natural reaction of STO to accommodate charge at the interface. Both effects have to be taken into account when comparing different samples grown by PLD. Annealing time and cooling rates as well as ambient annealing pressure are especially important for these processes, as they act not only as thermodynamic state variables but also control the equilibration kinetics.

## Methods

The experiments are divided into two parts. For both parts, LAO thin films are grown on TiO_2_ terminated STO (001) substrate (Crystec, GmbH) from single crystal LAO (001) (Crystec, GmbH) target using PLD with a 248 nm KrF excimer laser and a repetition frequency of 1 Hz. RHEED (kSA 400 system) is used to monitor the film growth. Clear intensity oscillations of the specular spot are observed as evidence for layer-by-layer growth. RHEED is also used for determining the number of deposited unit cells.

### Post Annealing Experiment

For the post annealing experiments (Fig. 1), 25 unit cells LAO have been grown on a 5 × 5 × 0.5 mm^3^ STO substrate at 973 K, a laser fluence of 1.89 J/cm^2^ and an oxygen pressure of 1 × 10^−4^ mbar (growth rate 40 ± 2 pulses/u.c.). After being cooled down to 473 K with a ramp rate of 10 K/min under 1 × 10^−4^ mbar oxygen pressure with UHV background of 10^−8^ mbar, the sample is cut into five pieces of 5 × 1 × 0.5 mm^3^. One sample is taken as as-grown reference. The other samples are then post-annealed at 973 K for 1 h in 10^−2^ mbar, 10^−3^ mbar and 10^−4^ mbar of oxygen. Low temperature resistivity measurements are then performed at variable temperature from 2 K to 300 K at a Physical Property Measurement System (PPMS^®^, Quantum Design Inc.).

### *In-Situ* Annealing Experiment

For the *in-situ* annealing experiment (Fig. 2), the deposition of LAO on 5 × 5 × 0.5 mm^3^ samples are carried out with laser fluence of 1.4 J/cm^2^ at 1073 K under 1 × 10^−3^ mbar (growth rate 42 ± 2 pulses/u.c.) and 1 × 10^−4^ mbar (growth rate 45 ± 2 pulses/u.c.) oxygen pressure with UHV background of 10^−8^ mbar. Note that the different laser fluence results from independent parameter optimization for each individual PLD setup in use. For 1 × 10^−3^ mbar, the thickness of the LAO films is 8 u.c., while for 1 × 10^−4^ mbar, the thickness is 5 u.c. (in order to enable XPS characterization). After switching off the ablation laser pulse for deposition, the sample is kept at the deposition temperature for different durations. Subsequently, the samples are quenched down by switching off the heating laser. Both sequences of LAO/STO films are characterized with room temperature Hall measurements in an 8404 HMS^®^ (Lakeshore Inc.). The post-annealed samples grown at 1 × 10^−4^ mbar are also characterized by *in-situ* X-ray Photoelectron Spectroscopy (XPS) before being removed from UHV conditions. The XPS measurements are performed with a PHI 5000 Versa Probe (Physical Electronics Inc., USA) with Al Kα X-ray illumination, a pass energy of 29.35 eV and at a photoemission angle of 45° using electron neutralization.

## Additional Information

**How to cite this article**: Xu, C. *et al.* Disentanglement of growth dynamic and thermodynamic effects in LaAlO_3_/SrTiO_3_ heterostructures. *Sci. Rep.* **6**, 22410; doi: 10.1038/srep22410 (2016).

## Supplementary Material

Supplementary Information
