# Supplementary material for: Disentanglement of growth dynamic and thermodynamic effects in LaAlO3/SrTiO3 heterostructures
Source: Sci Rep. 2016 Mar 24;6:22410. doi: 10.1038/srep22410 (PMC4806350; doi:10.1038/srep22410)
Supplement: Supplementary Information [file srep22410-s1.pdf]

Supporting Information:

Disentanglement of growth dynamic and thermodynamic effects in $LaAlO_3/SrTiO_3$ heterostructures

Chencheng Xu[a], Christoph Bäumer[a], Ronja Anika Heinen[a], Susanne Hoffmann-Eifert[a], Felix Gunkel[a,b], Regina Dittmann[a]*

[a]Peter Grünberg Insitut 7, Forschungszenturm Jülich GmbH, Jülich, Germany

[b]Insitut für Werkstoffe der Elektrotechnik II, RWTH Aachen University, Aachen, Germany

*Corresponding author: Chencheng Xu
Forschungszentrum Juelich GmbH
Peter Grünberg Institute 7 – Electronic Materials
52425 Juelich Germany
c.xu@fz-juelich.de

INTERPRETATION OF XPS SIGNAL The XPS measurements were carried out for the annealing time sample sequence grown and annealed at 1x 10^{-4} mbar as discussed in figure 2. Given the high surface sensitivity of XPS, a careful consideration of the probing depth is necessary to distinguish changes in the bulk of the sample from interface effects. With the kinetic energy of 1127 eV for Ti 2p3/2 photoelectrons, the inelastic mean free path λ is ~21.5 Å[1] (similar for both STO and LAO)[1], resulting in an effective λ_{eff} of ~21.5 Å for a photoemission angle of 0°. The photoelectron intensity contribution $I^{Ti2p}(z)$ generated at a depth z below the interface can then be related to the onsite photoelectron intensity $I_0{}^{Ti2p}(z)$ as

$$I^{Ti3+,2p}(z) = I_0^{Ti3+,2p}(z) \cdot exp(-\frac{z}{\lambda_{eff}}),$$

which makes the total photoelectron intensity a summation of the signals from all detectable cells. As a result, the probing depth of the XPS measurement (3 λ_{eff}) is ~65 Å and the information depth in STO is around 46 Å (~12 u.c. STO) with 19 Å (5 u.c.) LAO on top. As previous XPS and HAXPES studies of LAO/STO interfaces confirmed that the 2DEG is confined to a few unit cells (u.c.) STO beneath LAO, this means that the signal we measured using XPS results from a superposition of the 2DEG (~1-5u.c.) and a significant contribution from the STO bulk underneath the 2DEG.[2] Assuming a thickness of 1 u.c. for demonstration purposes (the same description is valid for different thicknesses, compare figure 3 in ref [2]), the Ti^{3+} contribution can be described as

$$\frac{I^{Ti3+,2p}}{I^{Ti3+,2p}+I^{Ti4+,2p}} = \frac{\sum_{1u.c.} I_{0,2DEG}^{Ti3+,2p}(z)\cdot \exp(-\frac{z}{\lambda_{eff}}) + \sum_{2u.c.}^{12u.c.} I_{0,bulk}^{Ti3+,2p}(z)\cdot \exp(-\frac{z}{\lambda_{eff}})}{\sum_{1u.c.} (I_{0,2DEG}^{Ti3+,2p}(z)+I_{0,2DEG}^{Ti4+,2p}(z))\cdot \exp(-\frac{z}{\lambda_{eff}}) + \sum_{2u.c.}^{12u.c.} (I_{0,bulk}^{Ti3+,2p}(z)+I_{0,bulk}^{Ti4+,2p}(z))\cdot \exp(-\frac{z}{\lambda_{eff}})}$$

Since the probing depth is much lower than the thickness of bulk STO, we can assume the bulk Ti3+ concentration within probing depth as a constant. Together with the assumption that the Ti^{3+} concentration in the 2DEG is depth independent, the Ti^{3+} concentration can be simplified as

$$\frac{I^{Ti3+,2p}}{I^{Ti3+,2p}+I^{Ti4+,2p}} = \frac{0.42\cdot \frac{I_{0,2DEG}^{Ti3+,2p}}{(I_{0,2DEG}^{Ti3+,2p}+I_{0,2DEG}^{Ti4+,2p})} + 1.87\cdot \frac{I_{0,Bulk}^{Ti3+,2p}(t)}{(I_{0,Bulk}^{Ti3+,2p}(t)+I_{0,Bulk}^{Ti4+,2p}(t))}}{0.42+1.87}$$

Thus we can describe the annealing-time-dependent XPS signal with a Ti^{3+} concentration of 18%/u.c. within the first STO unit cell at the interface for the 2DEG, which does not change with annealing time, and a bulk Ti^{3+} concentration of 1.6%/ u.c., which decay to zero with increasing annealing time. The charge carrier density of the sample annealed for 3500s is n_s ~1.2x10^{14}cm^{-2} (Figure 2(a)), which corresponds to 18% e^-/u.c. and is in good accordance with the estimated Ti^{3+} concentration from the XPS data.

Taking into account the vastly different thicknesses of the interface and the bulk, this change in bulk Ti^{3+} concentration also accounts for the change in G_S and n_S, which is around two orders of magnitude. As a summary, we conclude that the conductive interface between LAO/STO with n_S ~10^{14} cm^{-2} remains unchanged during the post annealing process, while the reduced STO bulk underneath the 2DEG, which is responsible for the extremely high G_S and n_S before the post annealing, is gradually oxidized.

RESCALING OF THE ELECTRON MOBILITY TO HIGH TEMPERATURE To make the in-situ annealing data comparable to the thermally equilibrated state measured at high temperature like 1073 K, we rescaled the electron mobility with the temperature dependence of electron mobility in SrTiO3[3, 4]. For temperature between 873 K and 1573 K the electron mobility (μ) can be estimated as $\mu = 3.95\times10^4 \cdot (T/K)^{-1.62} cm^2/(V\cdot s)$ [4]. By replacing the room temperature mobility by this estimated mobility at high temperature of 1073 K, the conductance data for the anneal time sequence (Figure 2) can be rescaled and compared to the conductance from HTEC.

NON-LINEAR HALL EFFECT AND ELECTRON MOBILITY IN A TWO-CHANNNEL MODEL

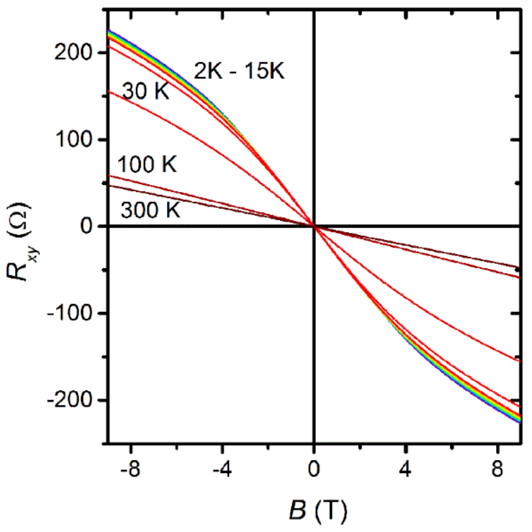

Figure 1 R_{xy}(B) for the as grown sample at different temperatures ranged from 2K to 300K.

Figure 1 shows Hall data recorded for the as-grown sample discussed in Fig. 1 of the main paper. Below 30K, $R_{xy}(B)$ shows a non-linear field dependence, which indicates a multi-channel conduction [5, 6] at the interface. Two electron conduction channels with distinguishable electron mobility are considered to fit this non-linearity. The corresponding electron mobility for each channel is displayed in Figure 2.The higher mobility is more than 2000 cm^2/Vs, while the lower mobility is around 300 cm^2/Vs.

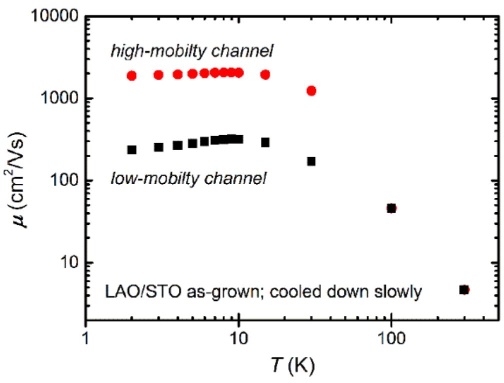

Figure 2 Electron mobility for (●) high mobility channel and (■) low mobility channel.

1. Tanuma, S., Powell, C. J. & Penn, D. R. Calculations of electron inelastic mean free paths. V. Data for 14 organic compounds over the 50–2000 eV range. Surface and Interface Analysis 21, 165-176 (1994).

2. Sing, M. et al. Profiling the interface electron gas of LaAlO3/SrTiO3 heterostructures with hard X-ray photoelectron spectroscopy. Phys. Rev. Lett. 102, 176805/1- (2009).

3. Moos, R., Menesklou, W. & Hardtl, K. H. Hall-mobility of undoped n-type conducting strontium-titanate single-crystals between 19K and 1373K. Appl. Phys. A-Mater. Sci. Process. 61, 389-395 (1995).

4. Moos, R. & Härdtl, K. H. Defect chemistry of donor-doped and undoped strontium titanate ceramics between 1000°C and 1400°C. J. Am. Ceram. Soc. 80, 2549-62 (1997).

5. Bell, C. et al. Dominant mobility modulation by the electric field effect at the LaAlO3/SrTiO3 interface. Phys. Rev. Lett. 103, 226802/1-4 (2009).

6. Fete, A. et al. Growth-induced electron mobility enhancement at the LaAlO3/SrTiO3 interface. Appl. Phys. Lett. 106, 51604/1-4 (2015).
